# Right ventricular systolic function and associated anatomic risk factors in fetuses with transposition of the great arteries: Evaluation by velocity vector imaging

**DOI:** 10.3389/fcvm.2022.973395

**Published:** 2023-01-10

**Authors:** Shan Lin, Haiyan Cao, Liu Hong, Xiaoyan Song, Kun Liu, Mingxing Xie, Yali Yang

**Affiliations:** ^1^Department of Ultrasound, Hubei No. 3 People’s Hospital of Jianghan University, Wuhan, China; ^2^Department of Ultrasound, Tongji Medical College, Union Hospital, University of Science and Technology, Wuhan, China; ^3^Hubei Province Clinical Research Center for Medical Imaging, Wuhan, China; ^4^Hubei Province Key Laboratory of Molecular Imaging, Wuhan, China

**Keywords:** complete transposition of the great arteries (d-TGA), Taussig-Bing anomaly (TBA), right ventricular function, velocity vector imaging, fetal echocardiography, anatomic risk factor

## Abstract

**Objectives:**

The aim of this study was to evaluate right ventricular (RV) systolic function in fetuses with transposition of the great arteries (TGA) using velocity vector imaging (VVI) and to investigate the impact of different factors on RV systolic function in TGA fetuses.

**Methods:**

This was a retrospective cross-sectional study of fetuses referred to our tertiary center between 2015 and 2019. Maternal and fetal baseline characteristics and conventional echocardiographic and myocardial deformation indices were collected in fetuses with TGA at 20–28 weeks’ gestation, which were compared with normal fetuses with comparable gestational age (GA). RV deformational parameters including global and regional longitudinal peak systolic strain, strain rate, and velocity were measured using off-line speckle tracking analysis. The univariate and multivariate linear regression analyses were established to evaluate the independent risk factors for RV global longitudinal systolic strain (RVGLSs) and strain rate (RVGLSRs).

**Results:**

In total, 78 fetuses with TGA [including 49 fetuses with complete transposition of the great arteries (d-TGA) and 29 fetuses with Taussig-Bing anomaly (TBA)] and 49 normal fetuses were included. Compared with normal controls, global and most regional RV longitudinal systolic peak velocity, strain, and strain rate were lower in d-TGA and TBA fetuses (*P* < 0.05). Compared with normal controls, global and most regional RV longitudinal systolic strain was lower in d-TGA fetuses without pulmonary stenosis (PS) and ventricular septal defect (VSD), while RVGLSs and RVGLSRs were lower in TBA fetuses without PS. The VSD was an independent determinant of RVGLSRs (*P* = 0.024) in the d-TGA group. Additionally, PS was an independent determinant of RVGLSs and RVGLSRs (*P* = 0.012, *P* = 0.027) in the TBA group.

**Conclusion:**

Early impairment of RV systolic function has already occurred in TGA fetuses during the 2nd trimester of pregnancy. PS, VSD, and foramen ovale (FO) were independent risk factors for decreased RV function.

## Introduction

The complete transposition of the great arteries (d-TGA) and Taussig-Bing anomaly (TGA-type double outlet right ventricle with subpulmonary ventricular septal defect, TBA) are both conotruncal abnormalities, characterized by complete septation but failed normal spiral of the great vessels. TBA and d-TGA both belong to the transposition of the great arteries (TGA) and share similar hemodynamics, clinical manifestations, and prognosis (1–3). In TGA fetuses, the aortic connects the right ventricle (RV), and the pulmonary artery connects the left ventricle (LV). Systemic and pulmonary circulation function as an invalid cycle to some extent and the blood is mixed oxygen blood, which may cause myocardial dysfunction, cardiac remodeling, fibrogenesis, and even heart failure (4). Therefore, it is worth exploring whether the fetuses with TGA have early impairment of right ventricular (RV) function. The early and sensitive assessment of the systolic RV function could provide important information for clinicians to make appropriate prenatal clinical decisions and early interventions after birth.

The RV is the main pumping ventricle of the TGA fetuses. It is critical to evaluate its systolic function. Velocity vector imaging (VVI) is an offline analysis software package that allows the evaluation of myocardial tissue motion and velocity without the limitations of Doppler echocardiography. The longitudinal systolic strain and strain rate are subsequently generated to evaluate fetal global and regional myocardial function (5–7). The aim of this study was to evaluate RV global and segmental cardiac function of the TGA fetuses in middle pregnancy using VVI and to investigate the impactors on their RV function. Therefore, the clinical significance of this study was the prenatal understanding of these combined malformations, which could provide some guidance for the fetal assessment of cardiac function as well as the intrauterine status.

## Materials and methods

### Study population

**FIGURE 1:**
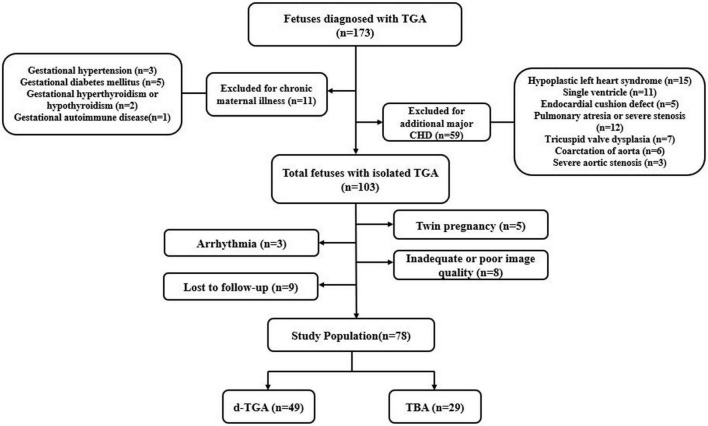
Flowchart of patient inclusion and exclusion criteria.

Fetuses with TGA at 20–28 weeks’ gestation that underwent prenatal echocardiography at our tertiary referral center (Union Hospital, Tongji Medical College, Huazhong University of Science and Technology) between August 2015 and November 2019 were included (Figure 1). They were classified into the following two subgroups: the d-TGA group and the TBA group. Cases with a pulmonary overriding of 50% and more were defined as d-TGA, whereas cases with a pulmonary overriding of less than 50% were defined as TBA. The diagnostic criteria for fetal pulmonary artery stenosis were the main artery or branches of the pulmonary artery with a *z*-score < −2. Each case was retrospectively identified through a review of the fetal echocardiography database of our center. Singleton fetuses with a structurally normal heart of comparable gestational age (GA) during the same study period were recruited as a normal group. Pregnancies complicated by chronic maternal disease (hypertension, diabetes mellitus, hyperthyroidism or hypothyroid, autoimmune disease, etc.), or fetuses with other major structural cardiac anomalies, extracardiac, or chromosomal abnormalities were excluded from the study. Other exclusion criteria were fetal arrhythmia, intrauterine growth retardation, twin pregnancy, poor image quality, and loss of follow-up. This study was approved by the Ethics Committee of Tongji Medical College of Huazhong University of Science and Technology.

### Fetal echocardiograms

Fetal echocardiograms were performed using a Voluson E8 or E10 (GE Healthcare, Zipf, Austria) ultrasound machine, equipped with an RM6C or RAB4-8-D probe. All ultrasound examinations were carried out according to the International Society of Ultrasound in Obstetrics and Gynecology (ISUOG) practice guidelines (8). Two-dimensional (2D) echocardiographic data including fetal growth measurements and conventional cardiac parameters (Table 1) were acquired during the scan. The 2D echocardiographic images of the four-chamber cardiac clips were stored with a frame rate of 60–80 frames/s. Additionally, the RV endocardial border must be clearly displayed. At least three complete cardiac cycle images were recorded and stored for offline analysis and processing.

**TABLE 1:** Baseline characteristics of the fetal study population.

Characteristics	Control (*n* = 49)	d-TGA (*n* = 49)	TBA (*n* = 29)	*P*
Maternal age, year	28.14 ± 3.93	29.00 ± 5.20	27.41 ± 3.20	0.281
AUA, week	25.09 ± 1.13	24.52 ± 2.82	24.42 ± 1.84	0.129
BPD (cm)	6.21 ± 0.35	6.04 ± 0.83	6.12 ± 0.59	0.404
HC (cm)	23.05 ± 1.12	22.34 ± 2.69	22.25 ± 2.13	0.073
AC (cm)	20.71 ± 1.17	20.25 ± 2.78	19.87 ± 2.29	0.143
FL (cm)	4.53 ± 0.30	4.40 ± 0.69	4.27 ± 0.48	0.036
EFW (g)	702 ± 105	684 ± 79	654 ± 75	0.124
**Pregnancy-labor history**				
Gravidity	2 (1–3)	2 (1–2)	2 (1–2)	0.745
Parity	0 (0–1)	0 (0–1)	1 (0–1)	0.186
Abortion	1 (0–1)	0 (0–1)	0 (0–1)	0.248
Fetal HR, bpm	148.90 ± 7.18	149.12 ± 8.43	146.38 ± 6.46	0.257
**Additional minor cardiac anomalies**				
PLSVC, *n* (%)	N/A	8 (16.3)	3 (10.3)	
ASA, *n* (%)	N/A	3 (6.1)	2 (6.9)	
Right-sided aortic arch, *n* (%)	N/A	6 (12.2)	3 (10.3)	
Aberrant subclavian artery, *n* (%)	N/A	4 (8.2)	2 (6.9)	
**Extracardiac abnormalities**				
Single umbilical artery, *n* (%)	N/A	3 (6.1)	2 (6.9)	0.195
Persistent right umbilical vein, *n* (%)	N/A	2 (4.1)	0 (0.0)	0.201

d-TGA, complete transposition of the great arteries; TBA, Taussig-Bing anomaly; AUA, average ultrasound age; BPD, biparietal diameter; HC, head circumference; AC, abdominal circumference; FL, femur length; EFW, estimated fetal weight; HR, heart rate; PLSVC, persistent left superior vena cava; ASA, atrial septal aneurysm.

### RV function analysis

**FIGURE 2:**
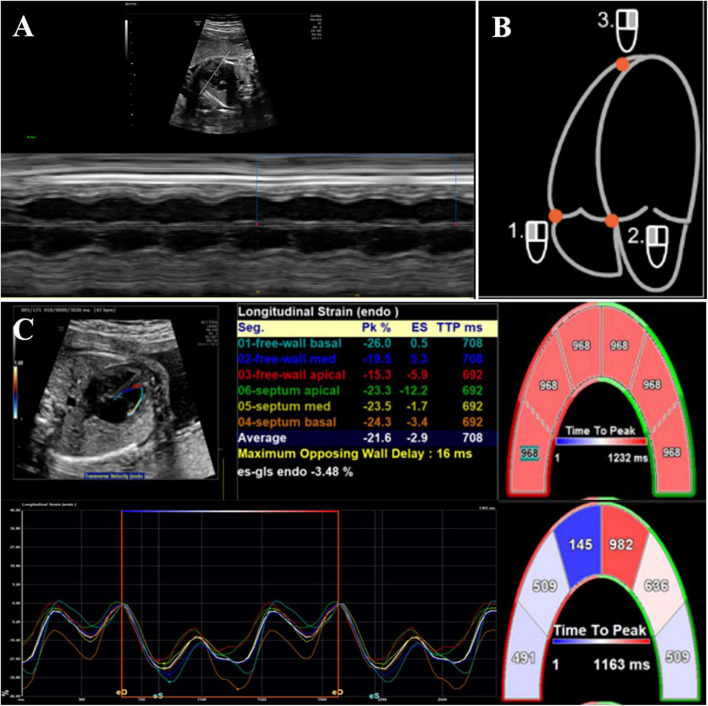
2D offline analysis of fetal myocardial deformation using velocity vector imaging. **(A)** Superimposed manual M-mode tracing of left ventricular wall motion, used as a surrogate for fetal electrocardiographic R-wave to calculate fetal heart rate, **(B)** the right ventricular endocardial boundary was manually traced by three-point method, and **(C)** global and regional longitudinal strain values/curves were generated automatically. Each curve demonstrated segmental strain and its average value.

For offline postprocessing, syngo VVI (version 2.3; Cardiac Performance Analysis, TomTec, Unterschleissheim, Germany) was used for both morphometric and deformation analyses. Image acquisition for deformation analysis was performed using a strict protocol. A 2D grayscale video clip (minimum of three clips for three to eight cardiac cycles) of an apical four-chamber view of the fetal heart was obtained. We used a narrower fan-shaped sampling frame and a single focus as much as possible to achieve a higher frame rate. We also used harmonic imaging when necessary to obtain the best contrast between the ventricular cavity and the endocardium. As fetal heart rate could not be displayed continuously because of the absence of a real-time fetal electrocardiogram (ECG), a superimposed manual M-mode tracing of the RV wall motion was used to define the cardiac cycle. We chose a still single frame, on which the endocardial interface was best visualized (usually mid-systole) (Figure 2A). The RV endocardial boundary was manually traced using the three-point method, with starting and ending points just below the tricuspid valve annulus (Figure 2B). Defining annuli and border position data were necessary components of the VVI algorithm. Images of the right ventricles were analyzed simultaneously from the same clip, and peak velocity, strain, and strain rate were then calculated automatically by the VVI software package (Figure 2C). Global and regional longitudinal systolic peak velocity, strain, and strain rate were then displayed automatically in a six-segment model for the RV. All calculations were repeated three times and then averaged.

### Intraobserver and interobserver reliability

Intraobserver variability for right ventricle mid-free wall longitudinal systolic peak velocity (RVLMV), right ventricle global longitudinal systolic strain rate (RVGLSs), and right ventricle basal septal longitudinal systolic strain rate (RV basal septal-SR) was assessed in 20 randomly chosen subjects (10 for TGA group and 10 for the control group) by a single investigator in the same cardiac cycle at a separate time several weeks apart. Interobserver measurements in the 20 randomly chosen subjects (10 for the TGA group and 10 for the control group) were performed by two reviewers on two separate occasions, each blinded to the other’s initial assessment.

### Statistical analysis

Statistical analyses were conducted using SPSS software (version 23.0; SPSS, Chicago, IL, USA). Descriptive data are presented as mean ± standard deviation (*SD*) for normally distributed variables, or median and interquartile range (IQR) for skewed distributions. For continuous variables, comparisons between groups were analyzed using one-way ANOVA and independent-sample test for normally distributed variables or Kruskal-Wallis test and adjusted *t*-test for skewed distributions. Univariate and multivariate linear regression analyses were performed to detect the determinants of RV global longitudinal systolic strain (RVGLSs) and RV global longitudinal systolic strain rate (RVGLSRs) in TGA fetuses. Intraclass correlation coefficients (ICCs) > 0.80 were considered excellent. Statistical significance was considered as *P* < *0.05*. Fetal cardiac *Z*-scores were obtained using a statistical program (Cardio Z; UBQO, Evelina Children’s Hospital, London, UK).

## Results

### Baseline characteristics and echocardiographic features

During the study period, a total of 127 fetuses were included in the analysis: 49 d-TGA, 29 TBA, and 49 GA-matched healthy controls. The baseline characteristics of the fetal study population are described in Table 1. TBA, d-TGA, and control groups were similar in gestational weeks (adjusted by ultrasound), maternal age, and pregnancy-labor history at the time of examination. Additional minor cardiac anomalies occurred in 21 (42.9%) d-TGA fetuses and 10 (34.5%) TBA fetuses, with persistent left superior vena cava (PLSVC), the most commonly seen. Extracardiac abnormalities including single umbilical artery and persistent right umbilical vein were observed in 5 (10.2%) d-TGA fetuses and 2 (6.9%) TBA fetuses.

Conventional echocardiographic parameters of the fetal study population are summarized in Table 2. Differences among d-TGA, TBA, and controls were found in *z*-scores of the aorta (AO), main pulmonary artery (MPA), left pulmonary artery (LPA), and AO/MPA ratio (*P* < 0.05). Compared to controls, d-TGA and TBA fetuses had wider aorta, while the pulmonary trunk was narrower in TBA compared with the other groups. The significant arterial discrepancy with an increased AO/MPA ratio was observed in TBA fetuses, indicating that the diameter of AO is greater than that of MPA, which was not detected in d-TGA fetuses. Ventricular dominance was not obvious in these groups. The conventional cardiac systolic function parameters (LVEF and RVFAC) did not differ between groups.

**TABLE 2:** Echocardiographic features of the fetal study population.

Parameter	Control (*n* = 49)	d-TGA (*n* = 49)	TBA (*n* = 29)	*P*
AO *z*-score	0.11 (−0.26 to 0.43)	0.63 (0.09–1.05)^†^	0.81 (−0.19 to 1.69)^†^	**0**.**001**
MPA *z*-score	0.51 (0.21–0.79)	1.13 (0.43–1.57)	−0.36 (−2.26 to 1.12)^†^*	**<0**.**001**
AO/MPA	0.80 (0.76–0.87)	0.83 (0.73–0.92)	1.09 (0.73–1.59)^†^*	**0**.**015**
LPA *z*-score	−0.03 (−0.33 to 0.33)	0.49 (−0.29 to 1.04)^†^	−0.03 (−1.15 to 0.39)*	**0**.**004**
RPA *z*-score	−0.10 (−0.56 to 0.25)	0.09 (−0.51 to 0.75)	−0.31 (−1.52 to 0.23)	0.058
Arterial duct *z*-score	−0.48 (−0.86 to −0.02)	−0.23 (−0.89 to 0.51)	−0.12 (−1.00 to 0.55)	0.399
LVEDD *z*-score	0.01 (−0.38 to 0.38)	0.08 (−0.71 to 0.66)	−0.26 (−0.91 to 0.54)	0.542
RVEDD *z*-score	−0.19 (−0.60 to 0.25)	−0.05 (−0.71 to 0.32)	0.14 (−0.68 to 0.59)	0.554
LVEDD/RVEDD	0.94 (−0.12 to 0.18)	0.96 (−0.32 to 0.45)	0.89 (−0.30 to 0.29)	0.168
LA/RA	0.90 ± 0.06	0.93 ± 0.10	0.86 ± 0.13*	0.069
LVEF (%)	55.38 ± 11.29	52.73 ± 9.30	51.06 ± 10.71	0.197
RVFAC (%)	36.91 ± 5.48	34.60 ± 7.39	33.95 ± 7.23	0.116
MR, *n* (%)	N/A	2 (4.1)	1 (3.4)	0.264
TR, *n* (%)	N/A	4 (8.2)	2 (6.9)	0.110

Data are expressed as mean ± SD or median (interquartile range). ^†^*P* < 0.05 vs. controls; *P < 0.05; d-TGA group vs. TBA group. d-TGA, complete transposition of the great arteries; TBA, Taussig-Bing anomaly; AO, aorta; MPA, main pulmonary artery; LPA, left pulmonary artery; RPA, right pulmonary artery; LVEDD, left ventricular end-diastolic diameter; RVEDD, right ventricular end-diastolic diameter; LA, left atrium; RA, right atrium; LVEF, left ventricle ejection fraction; RVFAC, right ventricle fractional area change; MR, minor or moderate mitral regurgitation; TR, minor or moderate tricuspid regurgitation.

The bold values indicate the statistical difference.

### RV deformation analysis using VVI

Right ventricular global and segmental deformation measures are shown in Table 3. Compared with controls, the RV global longitudinal systolic strain (GLSs), strain rate (GLSRs), and peak velocity (GLVs) were all lower in d-TGA and TBA fetuses (*P* < 0.01). In regional analysis, it also demonstrated impaired strain and strain rate in every segment of RV (*P* < 0.05), whereas the reduced systolic peak velocity was only observed in basal segments and mid septum. However, the global and regional deformation measures were similar between d-TGA and TBA (*P* > 0.05). It suggested that the RV systolic function in fetuses with d-TGA and TBA had already been impaired in the second trimester.

**TABLE 3:** Global and segmental deformation analysis of right ventricle.

VVI parameter		Control (*n* = 49)	d-TGA (*n* = 49)	TBA (*n* = 29)	*P*
Vs (cm/s)	Global	1.43 ± 0.35	1.07 ± 0.28^†^	1.05 ± 0.26^†^	**<0**.**001**
	Basal septal	2.19 ± 0.56	1.64 ± 0.52^†^	1.57 ± 0.42^†^	**<0**.**001**
	Mid septal	1.65 ± 0.44	1.40 ± 0.45^†^	1.37 ± 0.40^†^	**0**.**005**
	Apical septal	1.20 ± 0.37	1.19 ± 0.37	1.18 ± 0.44	0.989
	Apical free wall	1.26 ± 0.50	1.16 ± 0.44	1.08 ± 0.44	0.252
	Mid free wall	1.62 ± 0.44	1.42 ± 0.49	1.47 ± 0.55	0.095
	Basal free wall	2.15 ± 0.48	1.61 ± 0.60^†^	1.62 ± 0.58^†^	**<0**.**001**
Ss (%)	Global	−18.73 ± 3.69	−14.35 ± 3.20^†^	−13.04 ± 3.40^†^	**<0**.**001**
	Basal septal	−27.46 ± 10.29	−17.40 ± 6.81^†^	−21.40 ± 11.17^†^	**<0**.**001**
	Mid septal	−23.37 ± 9.70	−14.58 ± 5.89^†^	−16.61 ± 11.78^†^	**<0**.**001**
	Apical septal	−21.26 ± 8.89	−16.59 ± 6.17^†^	−15.78 ± 10.88^†^	**0**.**006**
	Apical free wall	−21.71 ± 8.24	−15.58 ± 5.23^†^	−17.14 ± 11.78	**<0**.**001**
	Mid free wall	−22.09 ± 9.12	−17.88 ± 8.70^†^	−17.78 ± 10.62	**0**.**047**
	Basal free wall	−27.66 ± 11.01	−16.88 ± 6.20^†^	−18.40 ± 8.10^†^	**<0**.**001**
SRs (/s)	Global	−1.64 ± 0.51	−1.45 ± 0.64^†^	−1.27 ± 0.48^†^	**0**.**003**
	Basal septal	−2.27 ± 1.05	−1.64 ± 0.58^†^	−1.84 ± 0.63^†^	**<0**.**001**
	Mid septal	−1.95 ± 0.63	−1.59 ± 0.67^†^	−1.72 ± 0.68^†^	**0**.**003**
	Apical septal	−1.94 ± 0.63	−1.63 ± 0.73^†^	−1.73 ± 0.58	**0**.**001**
	Apical free wall	−2.14 ± 0.84	−1.48 ± 0.42^†^	−1.64 ± 0.44^†^	**<0**.**001**
	Mid free wall	−1.95 ± 0.77	−1.57 ± 0.56^†^	−1.76 ± 0.80	**0**.**017**
	Basal free wall	−2.46 ± 0.84	−1.80 ± 0.91^†^	−1.63 ± 0.57^†^	**0**.**002**

Data are expressed as mean ± *SD*.

^†^*P* < 0.05 vs. controls.

d-TGA, complete transposition of the great arteries; TBA, Taussig-Bing anomaly; Vs, longitudinal systolic peak velocity; Ss, longitudinal systolic strain; SRs, longitudinal systolic strain rate.

The bold values indicate the statistical difference.

### RV function in d-TGA fetuses without pulmonary stenosis and ventricular septal defect and TBA fetuses without PS

In the d-TGA group, only 17 fetuses did not have a ventricular septal defect (VSD) and pulmonary stenosis (PS). Compared with controls, RVGLSs and most regional longitudinal systolic strain and strain rate were lower in d-TGA fetuses without VSD and PS (d-TGA-IVS-nPS group) (*P* < 0.05). However, RVGLSRs did not differ between those two groups (*P* > 0.05) (Table 4).

**TABLE 4:** Comparison of RV deformation parameters in d-TGA-nPS-IVS fetuses with control fetuses and TBA-nPS fetuses with control fetuses.

VVI parameter		d-TGA-nPS-IVS (*n* = 17)	Control (*n* = 49)	*P*	TBA-nPS (*n* = 14)	Control (*n* = 49)	*P*
Vs (cm/s)	Global	1.19 ± 0.25	1.43 ± 0.35	0.423	1.11 ± 0.29	1.43 ± 0.35	0.140
	Basal septal	1.80 ± 0.43	2.19 ± 0.56	0.105	1.48 ± 0.53	2.19 ± 0.56	**0**.**018**
	Mid septal	1.39 ± 0.41	1.65 ± 0.44	0.501	1.31 ± 0.44	1.65 ± 0.44	0.323
	Apical septal	1.24 ± 0.37	1.20 ± 0.37	0.805	1.24 ± 0.47	1.20 ± 0.37	0.866
	Apical free wall	1.18 ± 0.38	1.26 ± 0.50	0.716	1.09 ± 0.46	1.26 ± 0.50	0.765
	Mid free wall	1.32 ± 0.50	1.62 ± 0.44	**0**.**042**	1.49 ± 0.43	1.62 ± 0.44	0.762
	Basal free wall	1.55 ± 0.50	2.15 ± 0.48	**0**.**016**	1.66 ± 0.71	2.15 ± 0.48	**0**.**039**
Ss (%)	Global	−16.49 ± 1.94	−18.73 ± 3.69	**0**.**048**	−14.88 ± 3.71	−18.73 ± 3.69	**0**.**047**
	Basal septal	−20.26 ± 7.75	−27.46 ± 10.29	**0**.**033**	−16.89 ± 7.61	−27.46 ± 10.29	**0**.**003**
	Mid septal	−15.69 ± 6.74	−23.37 ± 9.70	**0**.**028**	−20.56 ± 15.04	−23.37 ± 9.70	0.613
	Apical septal	−16.86 ± 6.22	−21.26 ± 8.89	**0**.**024**	−14.14 ± 8.82	−21.26 ± 8.89	**0**.**022**
	Apical free wall	−15.64 ± 5.62	−21.71 ± 8.24	**0**.**036**	−15.41 ± 12.82	−21.71 ± 8.24	**0**.**028**
	Mid free wall	−19.42 ± 6.65	−22.09 ± 9.12	0.636	−19.44 ± 7.74	−22.09 ± 9.12	0.342
	Basal free wall	−19.42 ± 7.39	−27.66 ± 11.01	**0**.**014**	−20.64 ± 7.28	−27.66 ± 11.01	**0**.**037**
SRs (/s)	Global	−1.69 ± 0.96	−1.64 ± 0.51	0.885	−1.31 ± 0.35	−1.64 ± 0.51	**0**.**044**
	Basal septal	−1.69 ± 0.62	−2.27 ± 1.05	**0**.**015**	−1.89 ± 0.71	−2.27 ± 1.05	**0**.**049**
	Mid septal	−1.34 ± 0.90	−1.95 ± 0.63	**0**.**036**	−1.89 ± 0.92	−1.95 ± 0.63	0.867
	Apical septal	−1.61 ± 0.69	−1.94 ± 0.63	0.254	−1.64 ± 0.81	−1.94 ± 0.63	0.055
	Apical free wall	−1.38 ± 0.45	−2.14 ± 0.84	**0**.**012**	−1.60 ± 0.89	−2.14 ± 0.84	**0**.**029**
	Mid free wall	−1.55 ± 0.45	−1.95 ± 0.77	0.123	−1.66 ± 0.74	−1.95 ± 0.77	0.126
	Basal free wall	−1.81 ± 0.64	−2.46 ± 0.84	**0**.**013**	−1.49 ± 0.55	−2.46 ± 0.84	**0**.**015**

Data are expressed as mean ± *SD*.

d-TGA, complete transposition of the great arteries; TBA, Taussig-Bing anomaly; VSD, ventricular septal defect; IVS, intact interventricular septum; PS, pulmonary stenosis; Vs, longitudinal systolic peak velocity; Ss, longitudinal systolic strain; SRs, longitudinal systolic strain rate.

The bold values indicate the statistical difference.

Considering that all TBA fetuses were associated with VSD, we divided 29 TBA fetuses into the TBA-PS group (TBA with PS) and TBA-nPS group (TBA with well-developed pulmonary trunk), according to the presence of PS. Compared with the control group, RVGLSs, RVGLSRs, and most regional longitudinal systolic strain and strain rate in the TBA-nPS group were lower (*P* < 0.05), while other global and regional deformational parameters did not differ between groups (Table 4).

### Independent risk factors for RVGLSs and RVGLSRs in d-TGA or TBA fetuses

Clinical, anatomic, and ultrasonic variables, including maternal age, GA, associated cardiac abnormalities- PS, and VSD, the diameter of ascending aorta with a z-score < −2 (z-score of AO), the diameter of oval foramen with a z-score < −2 [z-score of foramen ovale (FO)], the diameter of the arterial duct with a z-score < −2 (z-score of DA), and the presence of mitral or tricuspid regurgitation (TR) are listed in Table 5. The correlation between these variables and RV strain parameters (RVGLSs and RVGLSRs) in d-TGA fetuses is examined using univariate and multivariate regression analyses, which revealed that PS, VSD, and z-score of FO were significantly correlated with RVGLSs (standardized β = 0.365, *P* = 0.018; standardized β = 0.496, *P* = 0.006, and standardized β = 0.328, *P* = 0.025, respectively). While, RVGLSRs were associated with maternal age, PS, and z-score of FO in univariate regression analysis. However, multivariate regression analysis indicated that the independent determinant of RVGLSRs was only VSD (standardized β = 0.328, *P* = 0.024) (Table 5).

**TABLE 5:** Univariate and multivariate regression analyses for RVGLS and RVGLSR in the fetuses with d-TGA.

Variables	RVGLS				RVGLSR			
	Univariate analysis		Multivariate analysis		Univariate analysis		Multivariate analysis	
	*r*-value	*P*-value	Standardized β	*P*-value	*r*-value	*P*-value	Standardized β	*P-*value
Maternal age (y)	<0.001	0.894			0.089	**0**.**052**		0.086
GA (AUA) (week)	0.004	0.605			0.016	0.328		
PS	0.126	**0**.**005**	0.365	**0**.**018**	0.068	**0**.**069**		0.127
VSD	0.254	**<0**.**001**	0.496	**0**.**006**	0.185	**0**.**003**	0.328	**0**.**024**
*z*-score of DA	<0.001	0.964			<0.001	0.932		
*z*-score of FO	0.186	**0**.**018**	0.328	**0**.**025**	0.087	**0**.**082**		0.175
*z*-score of AO	0.003	0.766			<0.001	0.920		
MR	0.052	0.312			0.028	0.258		
TR	0.028	0.278			0.001	0.885		

d-TGA, complete transposition of the great arteries; RVGLS, global longitudinal systolic strain; RVGLSR, global longitudinal systolic strain rate; GA (AUA), ultrasonic gestational age; PS, pulmonary stenosis; VSD, ventricular septal defect; *z*-score of DA, the diameter of the arterial duct with a *z*-score < −2; *z*-score of FO, the diameter of oval foramen with a *z*-score < −2; *z*-score of AO, the diameter of ascending aorta with a *z*-score < −2; MR, minor or moderate mitral regurgitation; TR, minor or moderate tricuspid regurgitation.

The bold values indicate the statistical difference.

Based on univariate regression analysis, the risk factors for RVGLSs in TBA fetuses included PS and TR. In multivariate regression analyses after adjusting for all the possible risk factors, only PS was an independent factor for RVGLSs (standardized β = 0.368, *P* = 0.012). Univariate logistic regression analysis revealed that maternal age and PS were correlated with the RVGLSRs in TBA fetuses. After adjusting for these possible risk factors, multivariate regression analyses showed that PS was an independent determinant of RVGLSRs (standardized β = 0.295, *P* = 0.027) (Table 6).

**TABLE 6:** Univariate and multivariate regression analyses for RVGLS and RVGLSR in the fetuses with TBA.

Variables	RVGLS				RVGLSR			
	Univariate analysis		Multivariate analysis		Univariate analysis		Multivariate analysis	
	*r*-value	*P*-value	Standardized β	*P-*value	*r*-value	*P*-value	Standardized β	*P-*value
Maternal age (y)	0.006	0.563			0.087	**0**.**069**		0.128
GA (AUA) (week)	0.005	0.685			0.008	0.526		
PS	0.356	**<0**.**001**	0.368	**0**.**012**	0.298	**0**.**005**	0.295	**0**.**027**
*z*-score of DA	0.002	0.879			<0.001	0.912		
*z*-score of FO	0.016	0.262			0.005	0.724		
*z*-score of AO	0.003	0.725			0.005	0.720		
MR	0.012	0.286			0.028	0.164		
TR	0.128	**0**.**034**		0.083	0.068	0.112		

TBA, Taussig-Bing anomaly; RVGLS, global longitudinal systolic strain; RVGLSR, global longitudinal systolic strain rate; GA (AUA), ultrasonic gestational age; PS, pulmonary stenosis; *z*-score of DA, the diameter of the arterial duct with a *z*-score < −2; *z*-score of FO, the diameter of oval foramen with a *z*-score < −2; *z*-score of AO, the diameter of ascending aorta with a *z*-score < −2; MR, minor or moderate mitral regurgitation; TR, minor or moderate tricuspid regurgitation.

The bold values indicate the statistical difference.

### Intraobserver and interobserver variability

**FIGURE 3:**
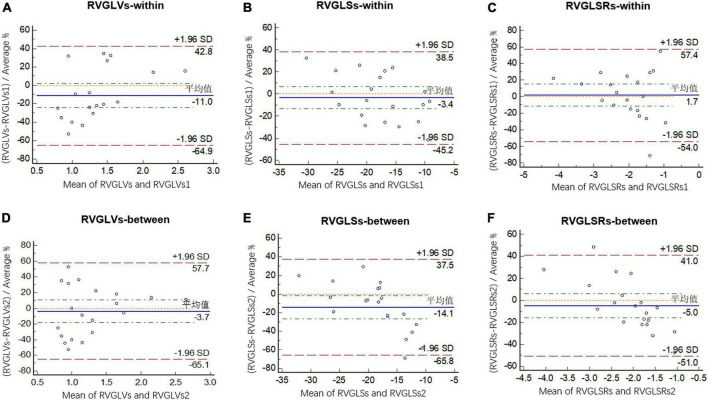
Bland-Altman analysis of intraobserver variability for RVGLVs **(A)**, RVGLSs **(B)**, and RVGLSRs **(C)** and interobserver variability for RVGLVs **(D)**, RVGLSs **(E)**, and RVGLSRs **(F)**. RVGLVs, right ventricle longitudinal systolic peak velocity; RVGLSs, right ventricle global longitudinal systolic strain; RVGLSRs, right ventricle global longitudinal systolic strain rate.

Figure 3 shows Bland-Altman plots of agreement between two separate measurements done by the first observer 4 weeks apart and between the measurements made by two observers. Intraclass correlation coefficients with 95% CIs showed moderate agreement for both intraobserver and interobserver comparisons (Table 7).

**TABLE 7:** Intraobserver and interobserver ICCs and 95% CI for right ventricular deformation parameters.

VVI parameter	Intra-observer		Inter-observer	
	**ICC**	**95%CI**	**ICC**	**95%CI**
RVGLVs (cm/s)	0.738	0.457–0.887	0.794	0.547–0.913
RVGLSs (%)	0.806	0.569–0.919	0.743	0.459–0.890
RVGLSRs (/s)	0.834	0.631–0.931	0.766	0.494–0.901

ICC, intraclass correlation coefficients; CI, confidence interval; RVGLVs, right ventricle longitudinal systolic peak velocity; RVGLSs, right ventricle global longitudinal systolic strain; RVGLSRs, right ventricle global longitudinal systolic strain rate.

## Discussion

The fetal blood circulation system is a parallel circulation, the left and right heart circulation are equally important, and the right heart advantage may be even more obvious in late pregnancy. In d-TGA and TBA fetuses, the right heart is connected to the aorta, thus, the right heart will withstand more pressure, especially after birth, the morphologically right ventricle is actually more similar to the strain pattern of the functional left ventricle at this point. It is probably more important to evaluate right heart function rather than left heart function. To the best of our knowledge, this study was the first to evaluate RV function using VVI in TGA fetuses and sought to investigate the correlation between RV cardiac function and different anatomic risk factors such as VSD or PS.

In our study, the maternal and fetal demographic characteristics, including maternal age, adjusted GA, estimated fetal weight, biparietal diameter, head circumference, abdominal circumference, femur length, pregnancy-labor history, fetal heart rate, and associated intracardiac/extracardiac anomalies, did not differ between groups. Consistent with previous literature, the most common intracardiac anomalies in TGA fetuses were VSD and PS (1). The *Z*-scores of AO, MPA, LPA, and AO/MPA ratio showed statistically significant differences between groups. The dimension of MPA was slightly wider than that of AO in normal fetuses; but in fetuses with TGA, not only the anatomical position of AO and MPA was changed, but also the stenosis of pulmonary trunk and branches were often detected (1, 9). A significant arterial discrepancy was observed in TBA fetuses, while ventricular disproportion was not obvious in our study. Moreover, the conventional cardiac function parameters such as LVEF and RVFAC presented slightly lower in d-TGA and TBA fetuses, without significant differences between groups. These results might indicate that, although the anatomy and hemodynamics of d-TGA and TBA fetuses were widely different from those of normal controls, no obvious changes were detected in ventricular morphological and conventional cardiac function indices in the second trimester (10, 11).

In our study, d-TGA and TBA fetuses had substantially lower values in global and most RV segmental longitudinal systolic peak velocity, strain, and strain rate compared with normal controls. These results indicated that RV function had already been impaired in mid-pregnancy, although the morphological features and traditional systolic function index (FAC) of RV were not obviously damaged.

This phenomenon might be related to the specific anatomical features of RV and insufficient coronary oxygen supply in TGA fetuses. First, unlike normal fetuses, the aortic artery did not arise from the left ventricle (LV) but RV in TGA, which made RV completely or partially replace the function of LV to bear the pressure of systemic circulation. In this condition, RV was more likely to act as a functional LV (11). As we know, RV had an irregular geometry with a thinner wall, which was mainly made of longitudinal fibers. It was characterized by weaker contractility, smaller afterload, and greater compliance when compared with LV. In addition, RV exhibited a peristaltic-like motion and did not have a torsional motion such as LV (12, 13). Therefore, myocardial damage and ventricular remodeling in RV became more possible when the RV volume load changed and the stress of the wall increased. Second, lower oxygen saturation in the aorta caused insufficient coronary oxygen supply in coronary arteries in TGA fetuses, even with some fetuses presenting abnormal development of coronary arteries. This could lead to myocardial hypoperfusion, which in turn resulted in myocardial ischemia and hypoxia in RV (14, 15). Persistent myocardial ischemia and hypoxia would contribute to ventricular remodeling. Such remodeling could also be developed in response to varied forms of myocardial injury and increased wall stress, causing reduced ventricular systolic function and eventually heart failure (16).

In d-TGA and TBA fetuses, systemic circulation depends on the right ventricle, while pulmonary circulation depends on the left ventricle. These two circulations work in parallel and communicate by three shunts, including FO, ductus arteriosus (DA), and VSD (17). In this study, we found that RV cardiac function was obviously decreased when d-TGA fetuses were combined with FO, VSD, or PS and TBA fetuses were combined with PS. Such anatomic features also proved to be the independent risk factors for impaired RV function. In fetal TGA with VSD, it presented a bidirectional shunt across VSD. The possible mechanism of its influence on RV function was mainly as follows: first, the presence of VSD led to an ineffective shunt during the systolic period, and it would cause increased myocardial work in systole, which could result in impaired myocardial function; second, the ineffective shunt between ventricles would result in RV volume overload, which might hinder the maturation of RV myocardium, impede the improvement of myocardial compliance, and slow the reduction of myocardial stiffness after birth (18, 19).

The independent determinant of RVGLSs included the diameter of oval foramen with a *z*-score < −2. This observation might reflect a smaller interatrial shunt with lower FO growth in fetuses with d-TGA, which might affect RV function. When d-TGA and TBA fetuses were combined with smaller FO, there might be a potential threat to fetal RV function. At the beginning of the second trimester, TGA fetuses had complex blood flow redistribution, which led to a lower FO growth, lower right-to-left shunt between the atria, and consequently, decreased antegrade ductal flow and increased pulmonary blood flow, and eventually, the decreased of arterial duct blood flow (17, 20). This would accelerate RV remodeling in TGA fetuses, resulting in myocardial damage.

When TGA fetuses were associated with dysplasia of the pulmonary trunk or branches, the main influencing mechanism on RV systolic function might be as follows: this study included fetuses with d-TGA and TBA fetuses with mild to moderate pulmonary stenosis, and although the oxygenated flow to the pulmonary artery was higher in TGA, the pulmonary flow might be significantly reduced caused by PS. It would result in fetal pulmonary circulation blood flow insufficiency and hypoxia (15, 21). Additionally, most of the blood enters the systemic circulation through the aorta and eventually returns to the right heart, causing a volume overload of RV. Ventricular wall stress also increased with sustained RV volume overload, leading to myocardial damage and ventricular remodeling (22, 23).

### Limitations

There were still some limitations in this study. First, this was a single-centered retrospective study. Some cases were excluded due to incomplete information, poor-quality/unavailable images, or extracardiac anomaly, and selection bias might occur. Second, the four-chamber view was the most amenable to longitudinal strain measurements, so we addressed only longitudinal deformation and did not evaluate circumferential or radial strain or twist or torsion. Finally, postnatal ventricular cardiac function in TGA cases was not analyzed in our study. Our further study might focus on the myocardial function changes after birth. In addition, given that the RV was the main pumping ventricle of the TGA fetuses, the left ventricular function would be further studied in the follow-up study.

## Conclusion

In TGA fetuses, impairment of RV function had already occurred in mid-pregnancy. PS and VSD were independent anatomic risk factors for decreased RV function. The deformation parameters might be more sensitive indicators for early impairment of fetal ventricular function than traditional cardiac function parameters.

## Data availability statement

The original contributions presented in this study are included in the article/supplementary material, further inquiries can be directed to the corresponding authors.

## Ethics statement

Written informed consent was not obtained from the individual(s) for the publication of any potentially identifiable images or data included in this article.

## Author contributions

SL and HC contributed to the conception of the study and manuscript writing. LH and XS contributed to the clinical data collection. KL contributed to data processing and statistical analysis. YY and MX proposed a critical revision to the manuscript. All authors contributed to this study and approved the submitted version.

## References

[1] Allen H Driscoll D Shaddy R. *Moss and Adams’ Heart Disease in Infants, Children, and Adolescents.* 8th ed. Baltimore, MD: Williams&Wilkins (2012). p. 1195–216.

[2] Rodríguez Puras M Cabeza-Letrán L Romero-Vazquianez M Santos de Soto J Hosseinpour R Gil Fournier M Mid-term morbidity and mortality of patients after arterial switch operation in infancy for transposition of the great arteries. *Rev Esp Cardiol.* (2014) 67:181–8. 10.1016/j.rec.2013.06.021 24774392

[3] Epstein J Franklin H. Cardiac development and implications for heart disease. *N Engl J Med.* (2010) 363:1638–47. 20961247 10.1056/NEJMra1003941

[4] Patterson A Zhang L. Hypoxia and fetal heart development. *Curr Mol Med.* (2010) 10:653–66. 10.2174/156652410792630643 20712587 PMC3075953

[5] Alsolai A Bligh L Greer R Gooi A Kumar S. Myocardial strain assessment using velocity vector imaging in normally grown fetuses at term. *Ultrasound Obstet Gynecol.* (2017) 52:352–8. 10.1002/uog.17549 28608400

[6] Eckersley L Howley L van der Velde M Khoo N Mah K Brooks P Quantitative assessment of left ventricular dysfunction in fetal Ebstein’s anomaly and tricuspid valve dysplasia. *J Am Soc Echocardiogr.* (2019) 32:1598–607. 10.1016/j.echo.2019.07.008 31551185

[7] Chelliah A Dham N Frank LH Mary D Anita K. Myocardial strain can be measured from first trimester fetal echocardiography using velocity vector imaging. *Prenat Diagns.* (2016) 36:483–8. 10.1002/pd.4813 26991266

[8] International Society of Ultrasound in Obstetrics and Gynecology, Carvalho JS Allan LD Chaoui R Copel JA DeVore G ISUOG practice guidelines (updated): sonographic screening examination of the fetal heart. *Ultrasound Obstet Gynecol.* (2013) 41:348–59. 10.1002/uog.12403 23460196

[9] Perez Delboy A Simpson L. Prenatal sonographic diagnosis of congenital heart disease and intrauterine growth restriction: a case control study. *J Clin Ultrasound.* (2007) 35:376–81. 10.1002/jcu.20308 17583562

[10] Itsukaichi M Kikuchi A Yoshihara K Serikawa T Takakuwa K Tanaka K. Changes in fetal circulation associated with congenital heart disease and their effects on fetal growth. *Fetal Diagn Ther.* (2011) 30:219–24. 10.1159/000330202 21849766

[11] Domínguez-Manzano P Mendoza A Herraiz I Escribano D Román V Aguilar J Transposition of the great arteries in fetal life: accuracy of diagnosis and short-term outcome. *Fetal Diagn Ther.* (2016) 40:268–76. 10.1159/000444296 26943122

[12] Sanz J Sánchez-Quintana D Bossone E Bogaard H Naeije R. Anatomy, function, and dysfunction of the right ventricle: JACC state-of-the-art review. *J Am Coll Cardiol.* (2019) 73:1463–82. 10.1016/j.jacc.2018.12.076 30922478

[13] Porayette P van Amerom J Yoo S Jaeggi E Macgowan C Seed M. MRI shows limited mixing between systemic and pulmonary circulations in fetal transposition of the great arteries: a potential cause of in utero pulmonary vascular disease. *Cardiol Young.* (2015) 25:737–44. 10.1017/S1047951114000870 24932863 PMC4411741

[14] Grotenhuis H Barbara C Mertens L Riessenkampff E Manlhiot M Seed M Left ventricular remodeling in long-term survivors after the arterial switch operation for transposition of the great arteries. *Eur Heart J Cardiovasc Imaging.* (2018) 20:101–7. 10.1093/ehjci/jey072 29800129

[15] Hauser M Bengel F Kuhn A. Myocardial blood flow and flow reserve after coronary reimplantation in patients after arterial switch and ross operation. *ACC Curr J Rev.* (2001) 103:1875–80. 10.1161/01.cir.103.14.1875 11294806

[16] Patten R Konstam M. Ventricular remodeling and the renin angiotensin aldosterone system. *Congest Heart Fail.* (2000) 6:187–92. 10.1111/j.1527-5299.2000.80159.x 12147951

[17] Lachaud M Dionne A Brassard M Charron MA Birca A Dehaes M Cardiac hemodynamics in fetuses with transposition of the great arteries and intact ventricular septum from diagnosis to end of pregnancy longitudinal follow-up. *Ultrasound Obstet Gynecol.* (2021) 57:273–81. 10.1002/uog.21920 31710736

[18] Miriam W Jonathon L Philip T Rekha R Shalan A Christopher RT Right ventricular assessment in adult congenital heart disease patients with right ventricle–to–pulmonary artery conduits. *J Am Soc Echocardiogr.* (2015) 28:522–32. 10.1016/j.echo.2014.11.016 25648672

[19] Chen J Xie L Dai L Yu L Liu L Zhou Y Right heart function of fetuses and infants with large ventricular septal defect: a longitudinal case-control study. *Pediatr Cardiol.* (2016) 37:1488–97. 10.1007/s00246-016-1462-z 27562129

[20] Vigneswaran T Zidere V Miller O Simpson J Sharland K. Usefulness of the prenatal echocardiogram in fetuses with isolated transposition of the great arteries to predict the need for balloon atrial septostomy. *Am J Cardiol.* (2017) 119:1463–7. 10.1016/j.amjcard.2017.01.017 28283176

[21] Berger-Kulemann V Berger R Mlczoch E Sternal D Mailath-Pokorny M Hachemian N The effects of hemodynamic alterations on lung volumes in fetuses with tetralogy of fallot: an MRI study. *Pediatr Cardiol.* (2015) 36:1287–93. 10.1007/s00246-015-1159-8 25894759

[22] Maeno Y Kamenir S Sinclair B van der Velde M Smallhorn J Hornberger L. Prenatal features of ductus arteriosus constriction and restrictive foramen ovale in d-transposition of the great arteries. *Circulation.* (1999) 99:1209–14. 10.1161/01.cir.99.9.1209 10069789

[23] Godfrey M Friedman K Drogosz M Rudolph A Tworetzky W. Cardiac output and blood flow redistribution in fetuses with D-loop transposition of the great arteries and intact ventricular septum: insights into pathophysiology. *Ultrasound Obstet Gynecol.* (2017) 50:612–7. 10.1002/uog.17370 27873373

